# Supplementary material for: Prediction of spallation induced transmutation rates for long-lived fission products via proton accelerator
Source: Sci Rep. 2026 Feb 12;16:8585. doi: 10.1038/s41598-026-38736-9 (PMC12976062; doi:10.1038/s41598-026-38736-9)
Supplement: Supplementary file 1 — Supplementary Information. [file 41598_2026_38736_MOESM1_ESM.pdf]

Supplementary Information

Table S1. Initial isotopic composition of tin (6.9 g/cm^3)[1].

Isotope	Half-Life (days)	Mass Fraction
Sn-115	stable	0.4%
Sn-116	stable	5.57%
Sn-117	stable	9.55%
Sn-118	stable	9.76%
Sn-119	stable	9.81%
Sn-120	stable	9.78%
Sn-122	stable	10.69%
Sn-124	stable	14.82%
Sn-126	8.4E+07	29.57%

Table S2. Initial isotopic composition of zirconium (6.5 g/cm^3)[1].

Isotope	Half-Life (days)	Mass Fraction
Zr-90	stable	1.73%
Zr-91	stable	13.34%
Zr-92	stable	16.22%
Zr-93	5.59E+08	20.26%
Zr-94	stable	21.97%
Zr-96	7.31E+21	26.48%

Table S3. Initial isotopic composition of selenium (4.3 g/cm^3)[1].

Isotope	Half-Life (days)	Mass Fraction
Se-77	stable	3.44%
Se-78	stable	6.53%
Se-79	1.08E+08	13.88%
Se-80	stable	21.31%
Se-82	stable	54.79%

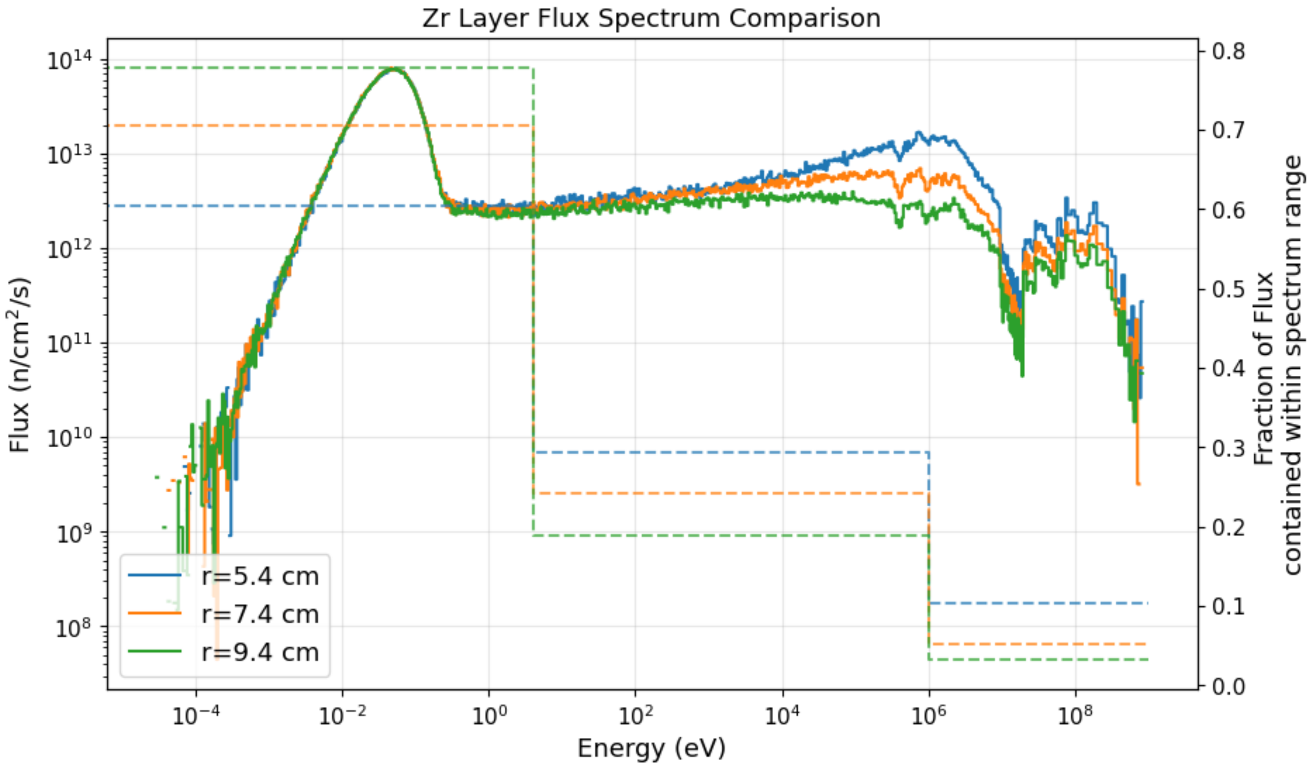

Figure S1. 708-group neutron flux spectrum in a pure D_2O system at different radii. The left axis is flux magnitude, and the right axis is the fraction of the total flux that falls within each energy range, the ranges being 0-4 eV (thermal), 4-1e6 eV (epithermal), and 1e6-1e9 eV (fast). All fluxes were tallied over equal-volume regions. These radii correspond to layers 0, 5, and 10 in Figure 2 of the main text.

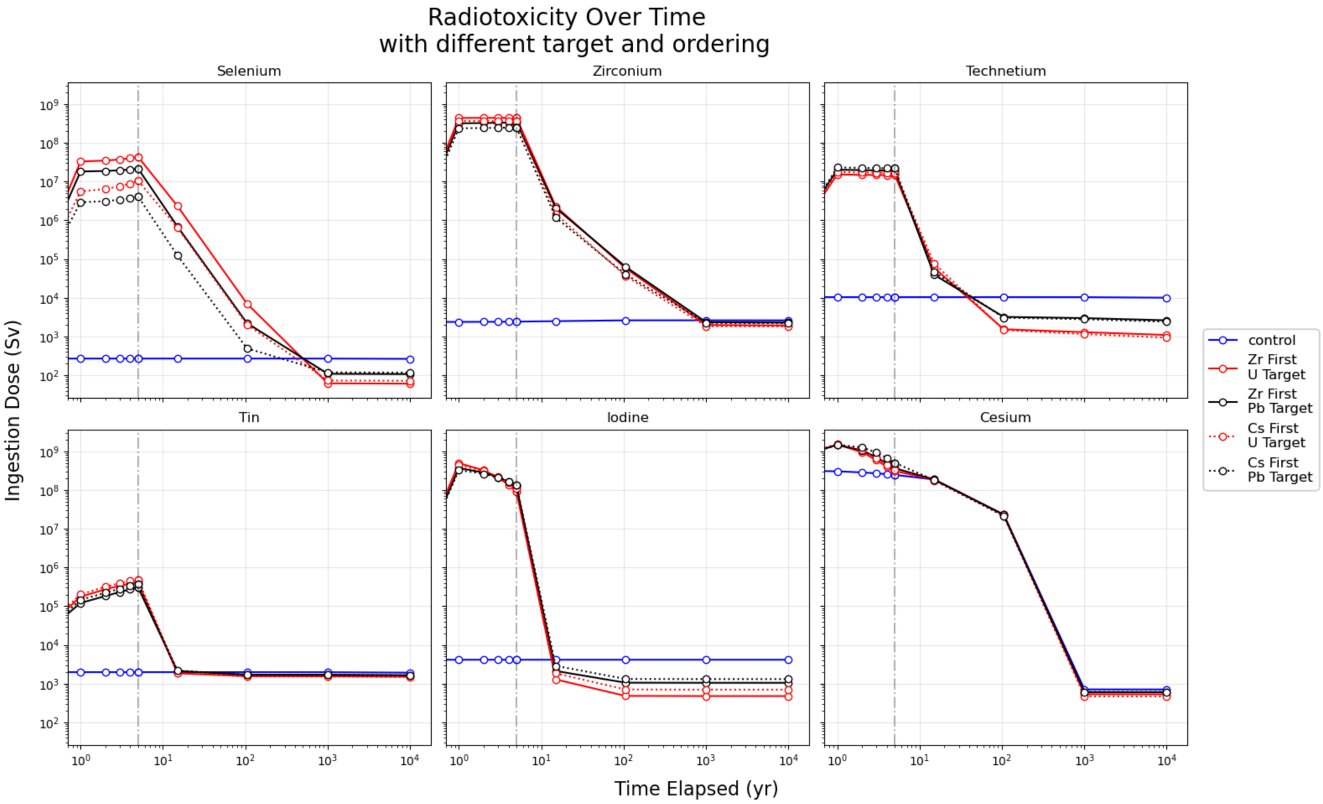

Figure S2. Radiotoxicity over time for each material in the combined geometry with different target material and ordering. The vertical line delineates when neutron transmutation ends. The control value is the radiotoxicity of the material over time under no neutron flux.

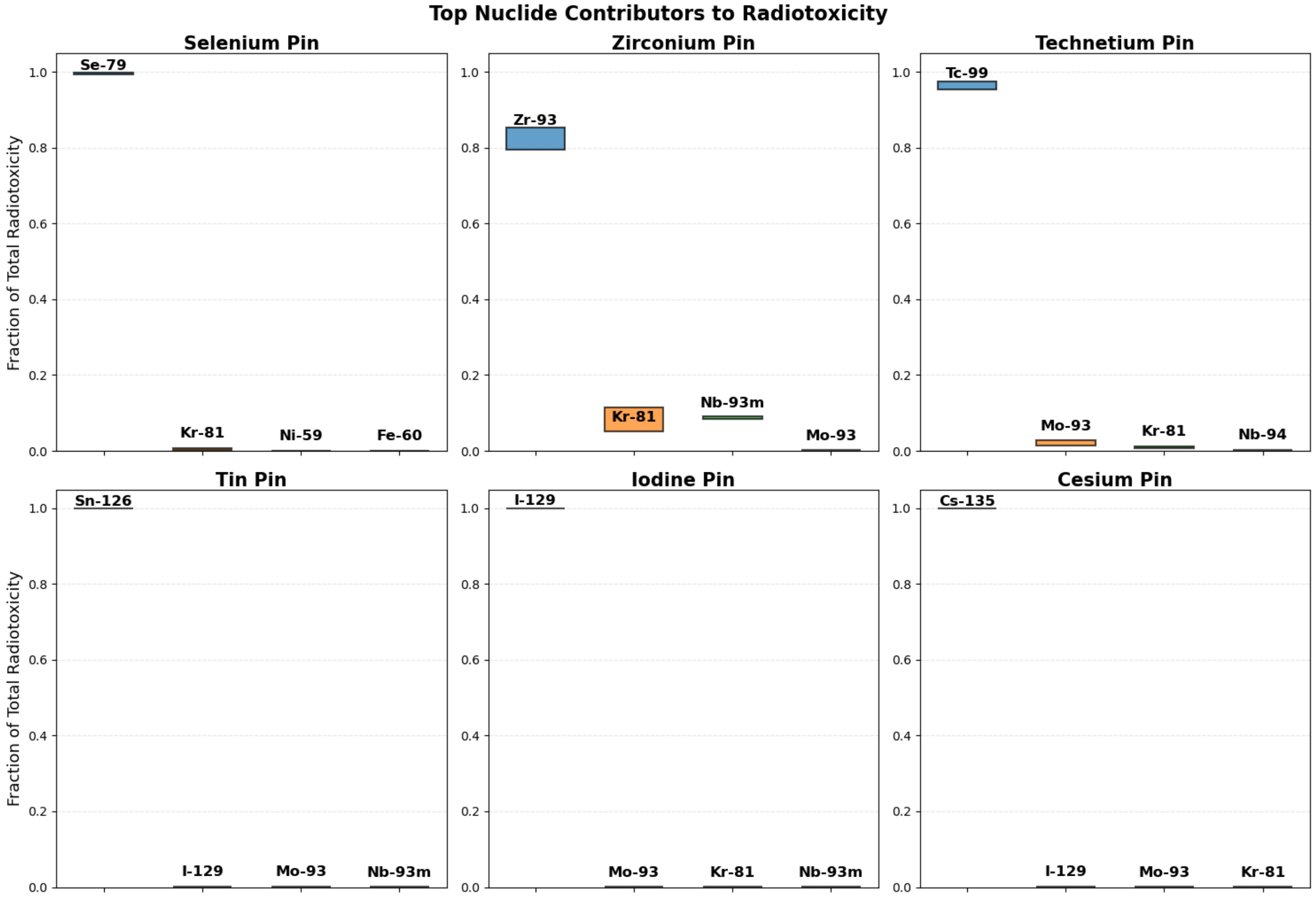

Figure S3. Fraction of the total radiotoxicity due to the top four contributing nuclides in the pin material. The width of each dataset represents the span of the values from different target material and element ordering. All values are from 5 years of irradiation and 1000 years of cooling.

Table S4. Initial isotopic composition of cesium (4.5 g/cm^3)[1].

Isotope	Half-Life (days)	Mass Fraction
Cs-133	stable	32.87%
Cs-134	754.32	0.33%
Cs-135	8.4E+08	38.06%
Cs-137	1.10E+04	28.74%

Table S5. Initial isotopic composition of iodine (4.5 g/cm^3)[1].

Isotope	Half-Life (days)	Mass Fraction
I-127	stable	27.85%
I-129	5.73E+09	72.15%

Target	Ordering	Element	Specific Ingestion Dose (Sv/g)	Relative Radiotoxicity compared to control
Pb	Zr 1st	Se	1.755E-2	0.404
		Zr	2.046E-2	0.894
		Tc	1.163E-1	0.288
		Sn	5.437E-1	0.860
		I	1.285E-1	0.254
		Cs	2.752E-2	0.849
	Cs 1st	Se	1.916E-2	0.441
		Zr	1.956E-2	0.855
		Tc	1.090E-1	0.270
		Sn	5.376E-1	0.851
		I	1.602E-1	0.317
		Cs	2.798E-2	0.863
U	Zr 1st	Se	9.985E-3	0.229
		Zr	1.732E-2	0.757
		Tc	5.042E-2	0.125
		Sn	4.982E-1	0.788
		I	5.833E-2	0.115
		Cs	2.427E-2	0.748
	Cs 1st	Se	1.176E-2	0.271
		Zr	1.609E-2	0.703
		Tc	4.506E-2	0.112
		Sn	4.892E-1	0.774
		I	8.543E-2	0.169
		Cs	2.139E-2	0.659

Table S6. Magnitude of radiotoxicity based on ingestion for each element, target material, and ordering. Values are after 5 years of irradiation and 1000 years of cooling. Relative value is in reference to the radiotoxicity of the material with no irradiation.

References

1. Wigeland, R. Nuclear fuel cycle evaluation and screening – final report. *Ida. Natl. Lab. Rep.* DOI: No.INL/EXT-14-31465 (2014).
